# Working together

**DOI:** 10.7554/eLife.69863

**Published:** 2021-06-08

**Authors:** Regan Odongo, Tunahan Çakır

**Affiliations:** Gebze Technical UniversityGebze, KocaeliTurkey

**Keywords:** systems biology, network analysis, whole-body modelling, Heart attack, multi-tissue, metabolically active tissues, Mouse

## Abstract

Heart attacks have a ripple effect on how other organs exchange biomolecules that help the heart respond to injury.

**Related research article** Arif M, Klevstig M, Benfeitas R, Doran S, Turkez H, Uhlén M, Clausen M, Wikström J, Etal D, Zhang C, Levin M, Mardinoglu A, Boren J. 2021. Integrative transcriptomic analysis of tissue-specific metabolic crosstalk after myocardial infarction. *eLife* **10**:e66921. doi: 10.7554/eLife.66921

A healthy heart relies on a close network of organs to work properly. Indeed, its cells require high levels of energy which is provided by biomolecules transported in the blood from other organs. If the blood supply is blocked, this can result in a heart attack which damages the heart’s muscle cells leading to the organ beating irregularly or, in some cases, failing all together. Our current understanding of heart attacks is limited to examining the alterations to tissues in the heart, but less is known about what happens in other organs.

Recent advances in molecular methods have begun to shed light on this issue, showing, for instance, that gene expression in the spleen and liver of pigs is significantly modified following a heart attack (Zimmermann et al., 2017). Computer simulations can predict the molecular alterations taking place during complex diseases, and could be harnessed to examine how different tissues respond to a heart attack (Priest and Tontonoz, 2019). Now, in eLife, Adil Mardinoglu (Royal Institute of Technology), Jan Boren (Sahlgrenska University Hospital) and colleagues – including Muhammad Arif and Martina Klevstig as joint first authors – report how they used this method, and other techniques, to investigate how heart attacks alter fat, skeletal muscle and liver tissues (Arif et al., 2021).

To identify which genes are altered in these tissues, the team (who are based in Sweden, Turkey and the United Kingdom) triggered acute heart attacks in mice by surgically blocking some of the arteries that supply blood to muscle cells in the heart (Figure 1). They then collected samples from the mice’s heart, skeletal muscle, fat tissue and liver, and measured how every single protein-coding gene was expressed using a highly sensitive technique called RNA sequencing. Comparing results from the heart attack model to tissue samples from control mice revealed striking changes in a number of genes, including four that had not previously been associated with this disease (*Flnc*, *Lgals3*, *Prkaca* and *Pprc1*). Most of these molecular changes took place after 24 hours, with the most profound effects occurring in the heart’s tissues.

**Figure 1.**
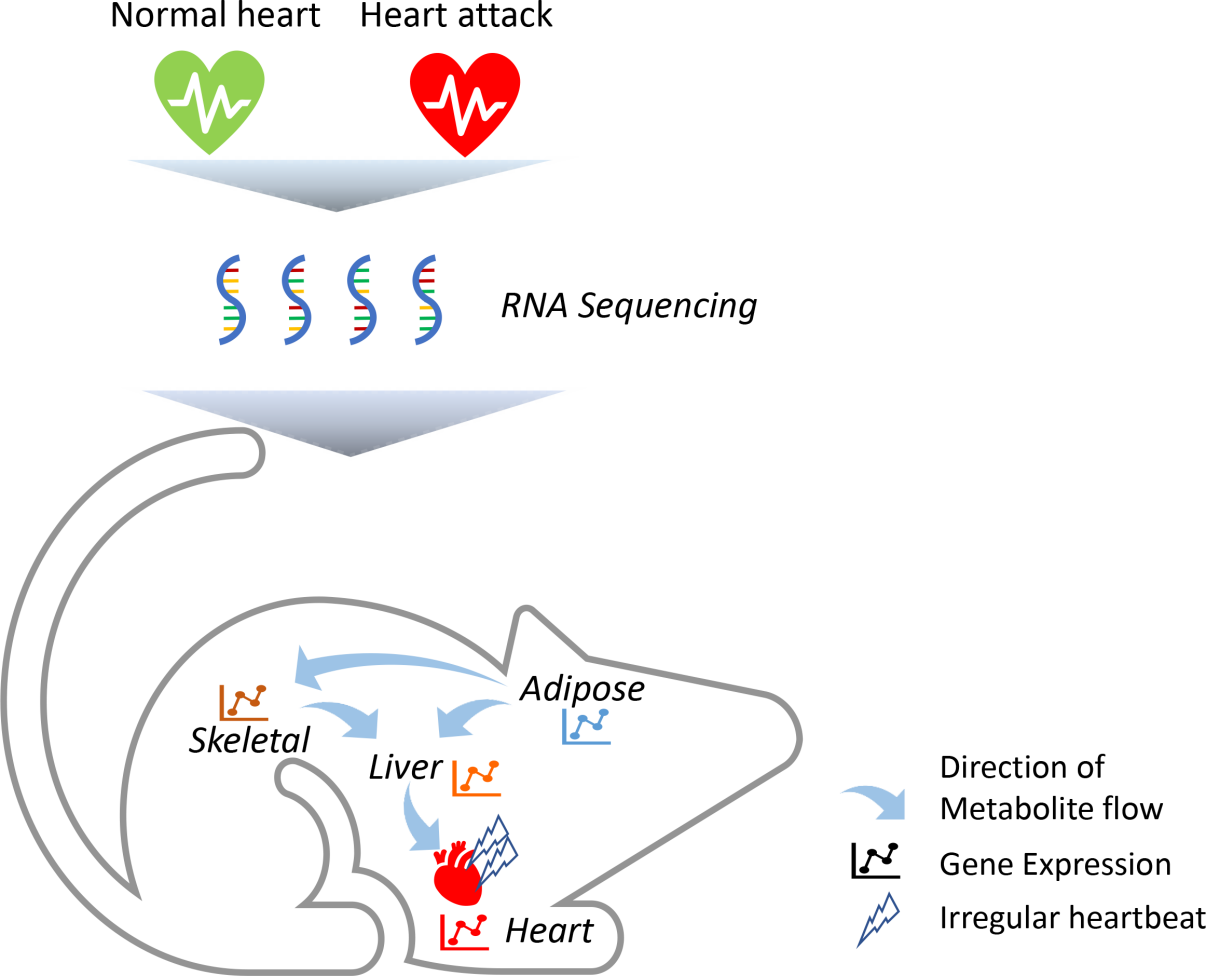
Heart attacks cause molecular changes in multiple tissues. Acute heart attacks were induced in mice by blocking some of the arteries that supply blood to the heart, resulting in an irregular heartbeat. Samples were then collected from the heart, skeletal muscle, liver and adipose, or 'fat', tissues of these mice (red heart) and control animals that had not undergone a heart attack (green heart). RNA sequencing revealed that the heart attack altered genes expressed in all four of these tissues. Further computational simulations suggest that these genetic changes alter the production of metabolites that are then taken up by the other tissues (represented by arrows).

Image credit: Regan Odongo.

Further analysis showed that genes associated with the immune system, RNA processes and mitochondrial function were commonly altered in the heart, skeletal muscle and fat tissues. Arif et al. also found groups of genes that were only modified in specific tissues. These unique clusters had a variety of roles, ranging from reacting to the heart attack to breaking down metabolites (biomolecules derived from glucose) so muscle cells in the heart can adequately respond to the injury. These findings suggest that the molecular changes induced by a heart attack have a ripple effect that changes how genes are expressed in other organs.

Next, Arif et al. performed computational simulations to predict which of the metabolites produced by these tissues might have an important role in supplying energy to the heart. To do this, they used a technique called reporter metabolite analysis which identifies key metabolites based on the genes expressed (Patil and Nielsen, 2005). Some of the metabolites were detected in all four tissues, while others were only found in either the heart, skeletal muscle, fat or liver. These metabolites were consistent with physiological changes commonly observed in patients that have experienced a heart attack (Doenst et al., 2013).

Advanced computational simulations were then conducted on a model in which all four tissues are linked together by blood vessels. This predicted that some of the metabolites synthesised by the liver are released into the blood and taken up by the heart’s tissues (Figure 1). The model also showed that some of the metabolites frequently found in the blood samples of heart attack patients are released from fat tissues and taken up by the liver and skeletal muscles. Taken together, these findings suggest that the heart, skeletal muscles, fat and liver all have a distinct molecular response to a heart attack. However, these genetic changes allow the tissues to ‘talk to each other’ and exchange metabolites that help the heart respond adequately to the damage caused by the heart attack.

This work provides novel insights into how spatially separated tissues with different embryonic origins can still coordinate after a heart attack. It also highlights how multi-tissue computational simulations can be used to unravel complex diseases. In future studies it will be critical to determine whether a similar multi-tissue crosstalk occurs in chronic heart attacks (which are more common), and if drugs that alter these molecular processes could improve the outcome for patients.
